# Ultra-Processing or Oral Processing? A Role for Energy Density and Eating Rate in Moderating Energy Intake from Processed Foods

**DOI:** 10.1093/cdn/nzaa019

**Published:** 2020-02-10

**Authors:** Ciarán G Forde, Monica Mars, Kees de Graaf

**Affiliations:** 1 Clinical Nutrition Research Center, A*STAR Singapore Institute for Clinical Sciences, Singapore; 2 Department of Physiology, Yong Loo Lin School of Medicine, National University of Singapore, Singapore; 3 Division of Human Nutrition and Health, Wageningen University and Research, Wageningen, Netherlands

**Keywords:** ultra-processed foods, unprocessed foods, food texture, energy density, eating rate, energy intake rate, obesity, metabolic disease

## Abstract

**Background:**

Recent observational data and a controlled in-patient crossover feeding trial show that consumption of “ultra-processed foods” (UPFs), as defined by the NOVA classification system, is associated with higher energy intake, adiposity, and at a population level, higher prevalence of obesity. A drawback of the NOVA classification is the lack of evidence supporting a causal mechanism for why UPFs lead to overconsumption of energy. In a recent study by Hall the energy intake rate in the UPF condition (48 kcal/min) was >50% higher than in the unprocessed condition (31 kcal/min). Extensive empirical evidence has shown the impact that higher energy density has on increasing ad libitum energy intake and body weight. A significant body of research has shown that consuming foods at higher eating rates is related to higher energy intake and a higher prevalence of obesity. Energy density can be combined with eating rate to create a measure of energy intake rate (kcal/min), providing an index of a food's potential to promote increased energy intake.

**Objective:**

The current paper compared the association between measured energy intake rate and level of processing as defined by the NOVA classification.

**Methods:**

Data were pooled from 5 published studies that measured energy intake rates across a total sample of 327 foods.

**Results:**

We show that going from unprocessed, to processed, to UPFs that the average energy intake rate increases from 35.5 ± 4.4, to 53.7 ± 4.3, to 69.4 ± 3.1 kcal/min (*P *< 0.05). However, within each processing category there is wide variability in the energy intake rate.

**Conclusions:**

We conclude that reported relations between UPF consumption and obesity should account for differences in energy intake rates when comparing unprocessed and ultra-processed diets. Future research requires well-controlled human feeding trials to establish the causal mechanisms for why certain UPFs can promote higher energy intake.

## Introduction

Recent observational studies suggest a relation between frequently consuming “ultra-processed” foods (UPFs), as defined by the NOVA classification, and the prevalence of obesity and related chronic diseases (1–3). Yet, despite numerous association studies, commentaries, and a single in-patient crossover feeding trial (4), the causal mechanisms behind these effects remain largely unclear (5). Early putative mechanisms for the increase in intake from UPFs include an unnaturally high sensory appeal or “hyperpalatability,” combined with a low satiety value (6). However, in the recent randomized controlled trial (RCT), experimental comparison of UPFs and unprocessed diets failed to confirm these proposed mechanisms, finding no reported differences in rated pleasantness or satiating value between the meals in either diet, despite a significantly increased energy intake in the UPF arm of the trial (4).

Findings from this in-patient feeding trial highlight potential risks of energy overconsumption and weight gain when consuming a diet dominated by UPFs (4). The study found that when participants were on the UPF diet arm they consumed an average of 508 kcal/d more energy than in the unprocessed arm, gaining an average of 0.9 kg during 2 wk with the UPF diet. This was reversed when they were returned to the unprocessed diet. The trial was designed as a comparison of energy intake on diets that differed in degree of processing, rather than a trial to identify the causal mechanism for increased consumption. Nevertheless, several factors may have contributed to the observed differences in energy intake, including differences in nonbeverage energy density, yet it currently remains unclear which element of the UPF diet was responsible for this large and consistent increase in energy intake and subsequent weight gain. The 2 diets were matched for the energy, macronutrients, salt, and fiber served; yet, with the exception of fasting peptide YY concentrations, there were negligible differences between the diets in endocrine or metabolic markers, such as satiety hormones and leptin or blood glucose and insulin concentrations. Both diets were, on average, equally liked and familiar and, despite differences in energy consumed, were equivalent for subjective satiety measures. However, the eating rate (g/min) or energy intake rate (kcal/min) during the ultra-processed diet was consistently and significantly higher for average gram per minute (37 vs 30 g/min) and >50% higher for average kilocalories per minute (48 vs 31 kcal/min), suggesting consistent differences in eating behaviors related to the higher energy intakes in the UPF arm [see Figure 2F in Hall et al. (4)].

Taken together, these findings suggest that 2 of the major determinants of ad libitum energy intake in the Hall et al. trial were the energy density and eating rate of the foods being consumed. Previous research by Rolls and colleagues has shown across many carefully designed studies how crucial energy density is in moderating daily energy intake [e.g., (7)]. Across dozens of experiments, controlling for many of the potential confounding factors in realistically designed test meals, Rolls and colleagues have shown repeatedly that higher-energy-density foods and diets promote greater energy intake and increased body weight, whereas lower-energy-density diets can support reductions in energy intake and body weight [see (8) for a review]. This appears to be true for men and women [e.g., (9)], for people with overweight and normal weight (7), for children and adults [e.g., (10)], and for the short term and long term [e.g., (11, 12)]. Recent findings suggest a tendency to underestimate the energy content of foods at higher energy densities, making it difficult to adjust intake and easier to consume more energy when food has a higher energy density (13). Consistent with this, the Rolls laboratory has shown that energy density prevails above macronutrient composition in driving higher energy intake. Whether energy is drawn from fat or carbohydrates, a higher energy density will always lead to increased energy consumption and longer-term increases in body weight (14). Whereas increased energy density promotes greater energy intake, by contrast, reducing energy density has been shown to reduce intake, with an effect that is sustained across several consecutive days with a lower-energy-dense diet (13).

In addition to energy density, an extensive body of research has shown the positive association between a higher eating rate and increased ad libitum energy intake. For example, ad libitum intake of chocolate milk was ∼30% higher than intake of a more viscous version with a similar macronutrient composition, energy density, and palatability. However, when eating rates of the 2 products were held constant, the differences in ad libitum intakes disappeared (15). Numerous experimental trials have confirmed the role of faster eating rates in promoting greater ad libitum energy intakes [e.g., (16–22)]. A systematic review and meta-analysis of 22 studies that measured both eating rate and intake concluded that higher eating rates were associated with higher ad libitum energy intakes (23). Research has demonstrated that food form and texture influence eating rate, with distinct differences in the rates at which liquid, semi-solid, and solid foods are consumed (17, 20, 24, 25). Within the recent Hall et al. RCT, the texture of the diets was not controlled and diets differed in nonbeverage energy density. A higher eating rate for liquid foods has been offered as one of the key ways in which sugar-sweetened beverages can promote increased energy intake (26). By contrast, harder solid food textures have been shown to reduce eating rate and ad libitum energy intake, demonstrating that the impact of eating rate on intake can be moderated by food texture (18, 19, 21, 22, 27, 28).

The current article provides a summary of findings to date that show how energy density and eating rate have been shown to influence energy intake, and the important role food texture plays in moderating this behavior. By pooling data from 5 previously published reports of food energy intake rates, we compared the energy intake rates for a large sample of foods from the United Kingdom, the Netherlands, Switzerland, and Singapore based on their degree of processing as defined by the NOVA classification. We propose that reported relations between UPF consumption and greater energy intake should account for differences in food energy intake rates when comparing unprocessed and ultra-processed diets. Finally, we outline the need for innovative food processing to reduce energy intake rates within the food supply, by reducing energy density and enhancing food texture to slow the rate of consumption.

## Methods

Eating rate has been objectively measured for a wide range of different meals and snacks across 5 independent studies, and these data formed the basis for the comparison of energy intake rates across different degrees of food processing (17, 20, 25, 29, 30). The study by Viskaal-van Dongen (17) measured the eating rate of 48 commonly consumed Dutch foods, which was later extended to include an additional 192 Dutch foods (29) to capture the foods that contribute most to energy intakes of Dutch adults based on the Dutch National Food Consumption Survey 2007–2010 (31). A study of 35 solid savory meal components was completed in Switzerland (20), and later complemented by a second study of 47 solid savory foods from Singapore (25). In both cases, the foods were chosen to represent a wide range of savory meal components that included common staple ingredients, meat, fish, fruits and vegetables, and snacks. This was further complemented by measured eating rates from a UK study that compared 20 popular commercially available prepackaged single-serve meals (30). Across all studies, the eating rate was measured using a similar approach and data were combined to represent a wide range of commonly consumed foods in the United Kingdom, Singapore, Switzerland, and the Netherlands (*n* = 330). Sampling focused on complete foods and snacks that are commonly consumed within meals, rather than ingredients, and produced a comprehensive set of food items for comparison. As a result, this sampling approach led to unbalanced groups and an overrepresentation of the UPF category in the final set, although this distribution reflects a similar distribution to that reported in everyday diets in the United States (32).

Energy intake rates were derived for each food by taking the measured eating rate (g/min) and multiplying this by the foods reported energy density in kilocalories per gram, resulting in a measure of energy intake rate (kcal/min). For each of the studies, eating rate was profiled objectively using a behavioral coding approach described previously (20), and energy density for each food was derived either from information on the pack, or the national food-composition tables as described in each of the studies (17, 20, 25, 29, 30).

The NOVA classification as defined by Monteiro et al. (6) was used to divide the foods into culinary ingredients, unprocessed foods, processed foods, and UPFs (6). Culinary ingredients were sugar, animal fats (butter) and vegetable oils, starches, salt, and vinegar, which were not the primary focus of the eating rates profiled in previous research. Three foods were outliers within their processing categories and were removed from the analysis to reduce the risk of skewing the comparison (apple juice in the unprocessed, breakfast drink in the processed, and chocolate milk in the UPF categories). Therefore, the final sample for comparison of energy intake rates across unprocessed, processed, and UPFs included *n* = 327 foods.

Unprocessed foods included dry, frozen or fresh fruits, vegetables, grains, or meats that had been subjected to minimal or no processing. UPFs included fresh meat, milk and plain yogurt, vegetables, eggs, legumes, fish and other seafood, and unsalted nuts and seeds. Fruit juice was described as unprocessed if it was freshly squeezed. Based on this designation, tea and coffee were classified as unprocessed and breads were unprocessed if they were homemade.

Processed foods were manufactured through the addition of culinary ingredients (i.e., fat, sugar, salt as described above) to natural fresh foods. Those foods included cheese; ham; salted, smoked, or canned meat or fish; pickled vegetables; salted or sugared nuts; beer; and wine.

UPFs were defined as industrial creations that contain ingredients not found in home cooking, in addition to culinary ingredients such as fat, sugar, and salt. UPFs included commercial breads (refined and whole grain), ready-to-eat breakfast cereals, cakes, sweet snacks, pizza, French fries, soft drinks (sodas and fruit drinks), ice cream, frozen meals and soups, whole-grain breads, commercial sweetened yogurts, commercial fruit juices, and ready-to-eat cereals.

The classification of foods into unprocessed, processed, and UPFs is not unequivocal and is highly dependent on the level of detail and available knowledge of the food ingredients and processing of each individual food item. The authors collated the available information for each food and made their own classification independently, and later discussed to reach a consensus on the final NOVA classification for each item. Any discordance for each classification was discussed to overcome any lack of detail in product description, unfamiliarity with ingredients/foods from different cultures, and clarity on the definitions between the different NOVA classifications. When these points were taken into account, the authors reached a final consensus on the NOVA classification for the 327 foods included in the final comparison listed alongside their measured energy intake rates (**Supplemental Table 1**).

## Results

The energy intake rate (kcal/min) of all foods was separated into tertiles from low to high (i.e., slow, medium, and fast energy intake rate) and frequencies for each processing classification are summarized in **Table 1**, alongside the percentage they represent from the total sample. Results illustrate a slightly higher proportion of UPFs in the higher-energy-intake-rate group, with slightly more unprocessed and processed foods in the lower-energy-intake-rate groups (Table 1).

**TABLE 1:** Frequencies and percentage of foods across energy intake rates (kcal/min) tertiles for unprocessed, processed, and ultra-processed foods^1^

	Low energy intake rate (tertile 1), *n* (%)	Medium energy intake rate (tertile 2), *n* (%)	High energy intake rate (tertile 3), *n* (%)
Unprocessed (*n* = 80)	53 (16)	17 (5.2)	10 (3)
Processed (*n* = 83)	29 (8.3)	33 (10.1)	21 (6.4)
Ultra-processed (*n* = 164)	27 (8.3)	61 (18.7)	76 (23.2)
Total (*n* = 327)	109	111	107

1 *n* = 327 foods split into tertiles based on their reported energy intake rates (kcal/min) and divided by their NOVA classification into unprocessed, processed, and ultra-processed. The percentage of the total sample (*n* = 327) is summarized in parentheses for each of the processing group × energy intake rate combination.

**FIGURE 1:**
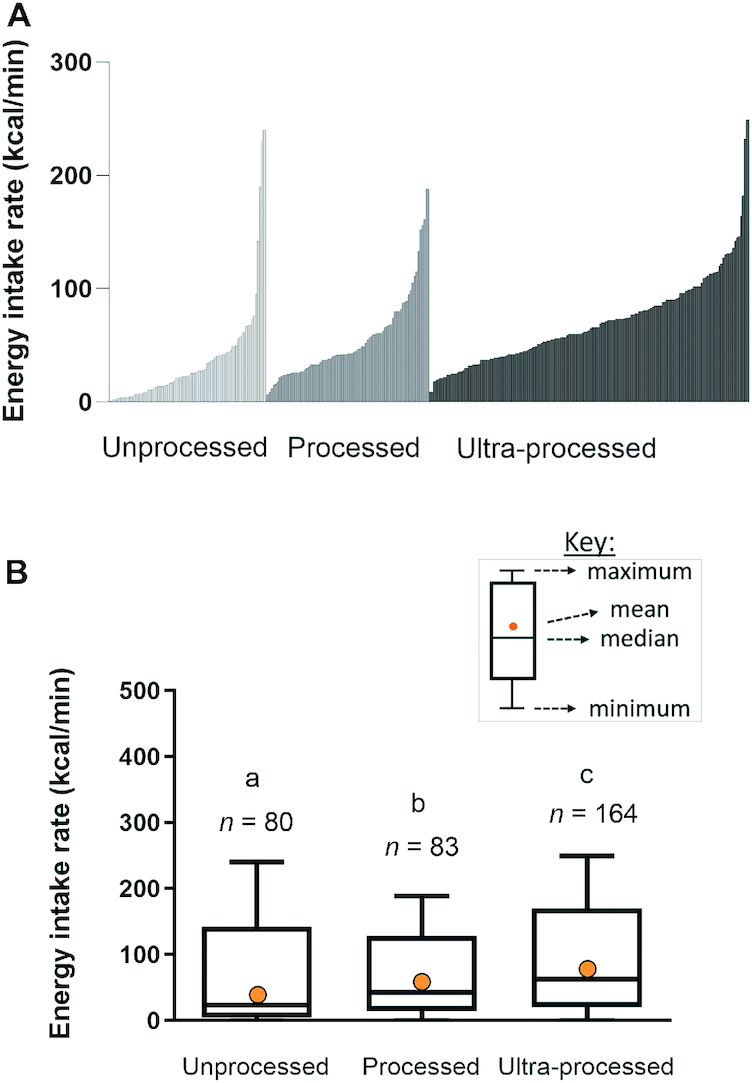
(A) Energy intake rates (kcal/min) ranked within each NOVA classification from low to high for the 3287 foods from unprocessed (light gray), processed (gray), and ultra-processed (black) foods separately. (B) Boxplot summary of the energy intake rate (kcal/min) for unprocessed, processed, and ultra-processed foods (1-factor ANOVA confirmed significant differences between categories, different letters indicate significant differences at *P *< 0.05; Least Significant Difference post hoc comparison).

**Figure 1**A summarizes the distribution of energy intake rates (kcal/min) across each of the processing classification groups for each food within each set. Figure 1B shows the mean, median, maximum, and minimum energy intake rates for unprocessed (*n* = 80), processed (*n* = 83), and UPFs (*n* = 164). The average energy intake rate (±SEM) for unprocessed foods in the current sample was 35.5 ± 4.4 kcal/min, but ranged from 2 kcal/min (bean sprouts/iceberg lettuce) to 230–240 kcal/min (fresh full-fat milk, orange juice). The processed foods had an average energy intake rate of 53.7 ± 4.3 kcal/min, but ranged from 6–7 kcal/min (pickled onion/canned mushroom) up to 188 kcal/min (skimmed milk). The UPFs in the current set had an average eating rate of 69.4 ± 3.1 kcal/min, but ranged at the lower end, from 0 kcal/min (Cola Light) to 9 kcal/min (powdered vegetable soup) up to 249 kcal/min for chocolate semi-skimmed milk. Differences in energy intake rates were significant across the 3 categories (*P* < 0.05): unprocessed, 35.5 (95% CI: 26.9, 44.2) kcal/min; processed, 53.7 (95% CI: 45.2, 62.2) kcal/min, and UPFs, 69.4 (95% CI: 63.3, 75.5) kcal/min.

Results indicate a wide distribution of energy intake rates (kcal/min) across all 3 processing groups, with a higher prevalence for higher energy intake rate foods among the UPFs, which was overrepresented among the foods sampled. However, the comparison in Figure 1A highlights an equivalently wide variability in energy intake rates within each processing classification group. Indeed, many of the unprocessed foods profiled had energy intake rates >100 kcal/min and equivalent to those observed in the UPF group. This demonstrates that higher energy intake rates are not the exclusive domain of highly processed foods, with higher energy intake rates seen across all processing designations.

## Discussion

The current comparison highlights that, across a wide sample of foods, those classified as UPFs had, on average, a faster energy intake rate (kcal/min) than unprocessed foods, although there was significant heterogeneity within each processing category. The UPF group was overrepresented in the current sample, making it an unbalanced comparison with fewer foods in the processed and unprocessed designations. Nevertheless, the current comparison highlights that previously reported relations between UPF consumption and increased energy intake and obesity prevalence may be confounded by underlying differences in the energy intake rates of the foods in these diets (4). Consumption of foods with a higher energy intake rate may offer a plausible mechanism by which increased UPF consumption could produce sustained increases in energy intake, and through this promotes longer-term overconsumption and higher body weight. This conclusion supports the recent findings from the RCT by Hall et al. (4), which showed a >50% higher energy intake rate within the UPF diet compared with the unprocessed diet, which was associated with increased calorie intake of >500 kcal/d (4). Taken together, the findings summarized in the current article suggest that combining the energy density and eating rate to estimate a food's energy intake rate (kcal/min) provides a useful index of the extent to which a food is likely to be consumed, and may offer an objective explanation for observed differences in ad libitum energy intake.

This finding is further supported by empirical data from numerous studies that show a direct impact of higher energy density and eating rate in increasing ad libitum energy intakes [i.e., (33)]. In addition, extensive evidence from population-based epidemiological studies of self-reported eating rates demonstrate a positive and sustained relation between eating faster, increased energy intake, and higher adiposity; BMI; and an elevated risk of metabolic disease (34–36). Previous research has suggested that a key element in the relation between texture, eating rate, and ad libitum energy intake is the orosensory exposure time taken during food oral processing (37, 38). Foods that require longer chewing and more time in the mouth for oral processing before swallowing are associated with higher expected satiation/satiety (20) and higher perceived fullness postconsumption (30, 39, 40). The time a food spends in the mouth during oral processing and the number of chews required per bite have been shown to have a direct effect on slowing energy intake rate and reducing ad libitum food intake (21, 22, 41–43).

The current discussion focuses on food properties such as texture, but it is important to acknowledge that eating rate is not only a property of the food but can also be considered a reflection of an individual's appetitive drive to eat. Research from the GEMINI twin cohort has shown that eating rate is a heritable trait that is positively associated with a higher BMI among children (44). Evidence from a prospective observational study in children of a high and low risk of obesity shows that a higher eating rate was predictive of increases in BMI between the ages of 4 and 6 y (45). Across a series of longitudinal observational studies with hundreds of children from the Growing Up in Singapore to Healthier Outcomes (GUSTO) cohort in Singapore, findings show that eating faster and for longer during a meal can lead to increases in ad libitum energy intake of ≤75% within a meal, and was associated with higher BMI and adiposity and faster increases in child adiposity over time (46–49). Children that eat faster tend to take larger bite sizes, fewer chews per bite (per kilocalories), and have a shorter orosensory exposure time per bite, in what has been described as an “obesogenic eating style” (47). At the level of the individual, eating rate has been shown to be a highly consistent behavior that is predictive of an individual's energy intake for successive consumptions of the same meal over consecutive weeks (50). Furthermore, research has shown that eating rates can be modified with training to slow energy intake rate and reduce the risk of overconsumption (51, 52).

The Hall et al. RCT did not profile postprandial endocrine responses, so it is currently unknown whether meals represented in the different test diets would produce significant changes in postprandial satiety hormone responses. Nevertheless, the lack of clear differences in fasting metabolic markers of food consumption between processed foods and UPFs from the Hall et al. study suggests that short-term increases in acute energy intake were more likely the result of behavioral processes, rather than an underlying disruption of metabolic regulation. The current article presents a comparison across a large and representative selection of the kind of energy densities and eating rates that are commonly encountered in the modern food environment, and suggests a wide diversity of energy intake rates that are likely to directly impact the propensity for a food to promote increased intake. Energy intake rates are rarely considered in the discussion on how certain foods can promote sustained increases in energy intakes, but may present a novel target for dietary intervention (53). To date, several acute (1-d) feeding trials have demonstrated the potential for slowing the energy intake rate to reduce ad libitum energy intakes by between 10% and 15% (22, 27). Early indications suggest this will be possible (52), and further research is required to test the longer-term efficacy of these approaches in controlled-feeding intervention trials, where reductions in a food's eating rate and energy density are manipulated to support reduced energy intake rate over time.

Modern food-processing techniques have received extensive criticism in recent years and are the central target of the NOVA classification system, which urges consumers to avoid or reduce their consumption completely. The NOVA classification system uses the term “processing” to refer to both product formulation and degree of processing, although it is likely the former will directly impact energy density, whereas the latter can impact composition, texture, and energy intake rate. Food processing is a broad term that consists of a wide variety of approaches for treating raw materials, such as grinding, milling, drying, cooking, frying, deboning, cutting, fermenting, freezing, pasteurizing, sterilization, and extrusion. These processes often break down the innate structures of the food, resulting in smaller particles or softer textures, and many of these processing steps are applied to improve the palatability, digestibility, and safety of the food and ensure it is suitable for consumption. Cooking has also been shown to significantly increase the availability of energy and micronutrients from meat and starch-rich foods in comparison to nonprocessed versions of the same ingredients (54). Food processes and formulations reduce the risk of harm to within an acceptable level (55), and within the last century food processing has contributed hugely to human health through the provision of safe, sustainable, edible, affordable, and palatable foods for billions of people across the world. Whereas food processing is often associated with the reduction in a food's innate structure (i.e., through mincing, chopping, grinding), processing can also be applied to add structure (i.e., extrusion, drying, baking) and, in combination with reformulation, can reduce a food's energy density and eating rate (5, 25). Food processing can also be applied to slow energy intake rates by adding texture or increasing volume and structure through processes such as extrusion, baking, and drying (25, 37, 56). One example of a UPF that has significantly reduced the contribution of added sugars to discretionary energy intake is the effective reformulation of sugar-sweetened beverages through the use of low and noncaloric sweeteners, which has been shown to reduce energy intake and body weight across a wide range of short- and long-term studies (57). Despite this, and based on the current NOVA classification guidelines, even reformulated products that provide little or no calories are still regarded as UPFs and their consumption is discouraged (58).

The application of food textures to moderate the rate and extent to which calories flow through our diets represents an opportunity to apply textures that can slow energy intake rates, and reduce the overall rate of calorie intake through modest changes to the food environment over time. The current comparison of energy intake rates is largely based on measured eating rates for individual meal components. A limitation of this comparison is the limited data on the eating rate of complex meals, with only a few examples to date (19, 22). Similarly, less is known about how the eating rate of individual meal components influences the eating rates and energy intake from composite mixed meals. Further research is required to better understand how manipulating food textures via food processing can be used to moderate habitual energy intake rates for the individual food items and meals that inform dietary patterns (28, 59). A deeper understanding of the relation between processing, texture, and oral processing behaviors could be used in the development of “design principles” to better control energy intake rates and provide guidance for product developers to reformulate food textures in a way that mitigates the risk associated with higher energy intake rates (35). Industrial food processing affords an important opportunity to apply wholesale changes to the forms and textures encountered in the food environment, and in combination with reformulation to reduce energy density, can be used to produce widespread improvements in the energy intake rates, palatability, and nutrient densities within the food supply (60). In addition to enhanced safety, shelf-life, nutrient density, and reduced energy intake rates, food processing is central to sustainable food production systems and will be needed to provide access to adequate nutrition for the future growth of the global population (61, 62).

The current article highlights a potential role for a new index—the “energy intake rate” (kcal/min) of a food—to help better explain the potential for a food to increase energy intakes and offers a potential mechanism for the previously observed increased energy intake from UPFs (4). The sensory properties of foods are rarely considered in the dietary recommendations that are made to reduce the risk of food-based chronic conditions such as obesity and/or type 2 diabetes; yet, they form an important connection between a food's form and nutrient composition, and the eating behaviors associated with increased intake (63). Future research should leverage the accumulated knowledge on how sensory cues such as food texture can be used to moderate the flow of calories through our diets.

Evidence has shown how both the energy density can be reduced and food texture can be manipulated to slow energy intake rates, providing food producers a previously unexplored opportunity for future food innovation and product renovation. Addressing the serious public health challenges posed by the modern food environment will require significant changes to our food systems and a series of objective criteria to identify and target the foods most in need of renovation and reformulation. The energy intake rate offers an objective, comparative approach to benchmarking the energy intake potential of foods within categories and across processing classifications and can help target those most likely to promote excessive consumption. Thereafter, the future challenge for food processors is to develop products that sustain consumer appeal with optimal satisfaction per kilocalorie consumed while reducing their potential to promote energy overconsumption.

## Supplementary Material

nzaa019_Supplement_Table

## Notes

CGF's role in the research was funded by the Biomedical Science Institute (Singapore) Food Structure Engineering for Nutrition and Health (H/18/01/a0/B11).

Author disclosures: CFG is on the Scientific Advisory Board for Kerry Health and Nutrition Institute, KdG is on the Scientific Advisory Board of Sensus, Roozendaal, and all authors have received reimbursements for speaking at meetings sponsored by companies that produce food and nutritional products. The authors report no conflicts of interest. The funding body had no role in the design or interpretation of the findings reported in the current manuscript.

Supplemental Table 1 is available from the “Supplementary data” link in the online posting of the article and from the same link in the online table of contents at https://academic.oup.com/cdn/.

## References

[1] Fiolet T, Srour B, Sellem L, Kesse-Guyot E, Allès B, Méjean C, Deschasaux M, Fassier P, Latino-Martel P, Beslay M Consumption of ultra-processed foods and cancer risk: results from NutriNet-Santé prospective cohort. BMJ. 2018;360:k322. 29444771 10.1136/bmj.k322 PMC5811844

[2] Schnabel L, Kesse-Guyot E, Allès B, Touvier M, Srour B, Hercberg S, Buscail C, Julia C Association between ultraprocessed food consumption and risk of mortality among middle-aged adults in France. JAMA Intern Med. 2019;179(4):490–8. 30742202 10.1001/jamainternmed.2018.7289 PMC6450295

[3] Srour B, Fezeu LK, Kesse-Guyot E, Allès B, Méjean C, Andrianasolo RM, Chazelas E, Deschasaux M, Hercberg S, Galan P et al. Ultra-processed food intake and risk of cardiovascular disease: prospective cohort study (NutriNet-Santé). BMJ. 2019;365:l1451. 31142457 10.1136/bmj.l1451 PMC6538975

[4] Hall KD, Ayuketah A, Brychta R, Cai H, Cassimatis T, Chen KY, Chung ST, Costa E, Courville A, Darcey V et al. Ultra-processed diets cause excess calorie intake and weight gain: an inpatient randomized controlled trial of ad libitum food intake. Cell Metab. 2019 Jul 2;30(1):67–77. 31105044 10.1016/j.cmet.2019.05.008 PMC7946062

[5] Gibney MJ, Forde CG, Mullally D, Gibney ER Ultra-processed foods in human health: a critical appraisal. Am J Clin Nutr. 2017;106(3):717–24. 28793996 10.3945/ajcn.117.160440

[6] Monteiro CA, Cannon G, Levy RB, Moubarac JC, Louzada ML, Rauber F, Khandpur N, Cediel G, Neri D, Martinez-Steele E et al. Ultra-processed foods: what they are and how to identify them. Public Health Nutr. 2019;22(5):936–41. 30744710 10.1017/S1368980018003762 PMC10260459

[7] Bell EA, Castellanos VH, Pelkman CL, Thorwart ML, Rolls BJ Energy density of foods affects energy intake in normal-weight women. Am J Clin Nutr. 1998;67(3):412–20. 9497184 10.1093/ajcn/67.3.412

[8] Rolls BJ The relationship between dietary energy density and energy intake. Physiol Behav. 2009;97(5):609–15. 19303887 10.1016/j.physbeh.2009.03.011 PMC4182946

[9] Kral TV, Roe LS, Rolls BJ Combined effects of energy density and portion size on energy intake in women. Am J Clin Nutr. 2004;79(6):962–8. 15159224 10.1093/ajcn/79.6.962

[10] Kling SM, Roe LS, Keller KL, Rolls BJ Double trouble: portion size and energy density combine to increase preschool children's lunch intake. Physiol Behav. 2016;162:18–26. 26879105 10.1016/j.physbeh.2016.02.019 PMC4899121

[11] Vernarelli JA, Mitchell DC, Rolls BJ, Hartman TJ Dietary energy density and obesity: how consumption patterns differ by body weight status. Eur J Nutr. 2018;57(1):351–61. 27738811 10.1007/s00394-016-1324-8

[12] Smethers AD, Roe LS, Sanchez CE, Zuraikat FM, Keller KL, Rolls BJ Both increases and decreases in energy density lead to sustained changes in preschool children's energy intake over 5 days. Physiol Behav. 2019;204:210–18. 30831180 10.1016/j.physbeh.2019.02.042 PMC6475467

[13] Brunstrom JM, Drake AC, Forde CG, Rogers PJ Undervalued and ignored: are humans poorly adapted to energy-dense foods?. Appetite. 2018;120:589–95. 29038018 10.1016/j.appet.2017.10.015

[14] Rolls BJ, Bell EA, Castellanos VH, Chow M, Pelkman CL, Thorwart ML Energy density but not fat content of foods affected energy intake in lean and obese women. Am J Clin Nutr. 1999;69(5):863–71. 10232624 10.1093/ajcn/69.5.863

[15] De Wijk RA, Zijlstra N, Mars M, De Graaf C, Prinz JF The effects of food viscosity on bite size, bite effort and food intake. Physiol Behav. 2008;95(3):527–32. 18721823 10.1016/j.physbeh.2008.07.026

[16] Hogenkamp PS, Stafleu A, Mars M, Brunstrom JM, de Graaf C Texture, not flavor, determines expected satiation of dairy products. Appetite. 2011;57(3):635–41. 21871509 10.1016/j.appet.2011.08.008

[17] Viskaal-van Dongen M, Kok FJ, de Graaf C Eating rate of commonly consumed foods promotes food and energy intake. Appetite. 2011;56(1):25–31. 21094194 10.1016/j.appet.2010.11.141

[18] Forde CG, Bolhuis D, Thaler T, Graaf CD, Martin N Influence of meal texture on eating rate and food intake: results from three ad-libitum trials. Appetite. 2013;71:474.

[19] Forde CG, van Kuijk N, Thaler T, de Graaf C, Martin N Texture and savoury taste influences on food intake in a realistic hot lunch time meal. Appetite. 2013;60:180–6. 23085683 10.1016/j.appet.2012.10.002

[20] Forde CG, van Kuijk N, Thaler T, de Graaf C, Martin N Oral processing characteristics of solid savoury meal components, and relationship with food composition, sensory attributes and expected satiation. Appetite. 2013;60(0):208–19. 23017464 10.1016/j.appet.2012.09.015

[21] Bolhuis DP, Forde CG, Cheng Y, Xu H, Martin N, de Graaf C Slow food: sustained impact of harder foods on the reduction in energy intake over the course of the day. PLoS One. 2014;9(4):e93370. 24695412 10.1371/journal.pone.0093370 PMC3973680

[22] McCrickerd K, Lim CM, Leong C, Chia EM, Forde CG Texture-based differences in eating rate reduce the impact of increased energy density and large portions on meal size in adults. J Nutr. 2017;147(6):1208–17. 28446630 10.3945/jn.116.244251

[23] Robinson E, Almiron-Roig E, Rutters F, de Graaf C, Forde CG, Tudur Smith C, Nolan SJ, Jebb SA A systematic review and meta-analysis examining the effect of eating rate on energy intake and hunger. Am J Clin Nutr. 2014;100(1):123–51. 24847856 10.3945/ajcn.113.081745

[24] Mourao DM, Bressan J, Campbell WW, Mattes RD Effects of food form on appetite and energy intake in lean and obese young adults. Int J Obes. 2007;31(11):1688–95. 10.1038/sj.ijo.0803667 17579632

[25] Forde C, Leong C, Chia-Ming E, McCrickerd K Fast or slow-foods? Describing natural variations in oral processing characteristics across a wide range of Asian foods. Food Function. 2017;8(2):595–606. 27883158 10.1039/c6fo01286h

[26] De Graaf C, Kok FJ Slow food, fast food and the control of food intake. Nat Rev Endocrinol. 2010;6(5):290–3. 20351697 10.1038/nrendo.2010.41

[27] Forde CG. From perception to ingestion; the role of sensory properties in energy selection, eating behaviour and food intake. Food Qual Preference. 2018;66:171–7.

[28] Wee MSM, Goh AT, Stieger M, Forde CG Correlation of instrumental texture properties from textural profile analysis (TPA) with eating behaviours and macronutrient composition for a wide range of solid foods. Food Function. 2018;9(10):5301–12. 30256358 10.1039/c8fo00791h

[29] van den Boer JHW, Werts M, Siebelink E, de Graaf C, Mars M The availability of slow and fast foods in the Dutch diet: the current situation and opportunities for interventions. Foods. 2017;6(10):87. 10.3390/foods6100087 PMC5664026 28974054

[30] Ferriday D, Bosworth ML, Godinot N, Martin N, Forde CG, Van Den Heuvel E, Appleton SL, Mercer Moss FJ, Rogers PJ, Brunstrom JM Variation in the oral processing of everyday meals is associated with fullness and meal size; a potential nudge to reduce energy intake?. Nutrients. 2016;8(5):315. 10.3390/nu8050315 PMC4882727 27213451

[31] van Rossum CT, Fransen HP, Verkaik-Kloosterman J, Buurma-Rethans EJ, Ocke MC Dutch National Food Consumption Survey 2007–2010: diet of children and adults aged 7 to 69 years. Amsterdam, The Netherlands: National Institute for Public Health and the Environment; 2011.

[32] Steele EM, Juul F, Neri D, Rauber F, Monteiro CA Dietary share of ultra-processed foods and metabolic syndrome in the US adult population. Prev Med. 2019;125:40–8. 31077725 10.1016/j.ypmed.2019.05.004

[33] Karl JP, Young AJ, Rood JC, Montain SJ Independent and combined effects of eating rate and energy density on energy intake, appetite, and gut hormones. Obesity. 2013;21(3):E244–52. 23592679 10.1002/oby.20075

[34] Ohkuma T, Hirakawa Y, Nakamura U, Kiyohara Y, Kitazono T, Ninomiya T Association between eating rate and obesity: a systematic review and meta-analysis. Int J Obes. 2015;39:1589–96. 10.1038/ijo.2015.96 26100137

[35] Teo PS, Forde CG The impact of eating rate on energy intake, body composition and health. In: Meiselman HL editor. Handbook of eating and drinking: interdisciplinary perspectives. Cham: Springer International Publishing; 2019, p. 1–27.

[36] van den Boer JH, Kranendonk J, van de Wiel A, Feskens EJ, Geelen A, Mars M Self-reported eating rate is associated with weight status in a Dutch population: a validation study and a cross-sectional study. Int J Behav Nutr Phys Act. 2017;14(1):121. 28886719 10.1186/s12966-017-0580-1 PMC5591506

[37] de Graaf C Texture and satiation: the role of oro-sensory exposure time. Physiol Behav. 2012;107(4):496–501. 22609070 10.1016/j.physbeh.2012.05.008

[38] Bolhuis DP, Lakemond C, De Wijk R, Luning P, De Graaf C Both longer oral sensory exposure to and higher intensity of saltiness decrease ad libitum food intake in healthy normal-weight men. J Nutr. 2011;141(12):2242–8. 22049294 10.3945/jn.111.143867

[39] Zhu Y, Hsu WH, Hollis JH The impact of food viscosity on eating rate, subjective appetite, glycemic response and gastric emptying rate. PLoS One. 2013;8(6):e67482. 23818981 10.1371/journal.pone.0067482 PMC3688614

[40] Zhu Y, Hsu WH, Hollis JH Increasing the number of masticatory cycles is associated with reduced appetite and altered postprandial plasma concentrations of gut hormones, insulin and glucose. Br J Nutr. 2013;110(2):384–90. 23181989 10.1017/S0007114512005053

[41] Zijlstra N, de Wijk RA, Mars M, Stafleu A, de Graaf C Effect of bite size and oral processing time of a semisolid food on satiation. Am J Clin Nutr. 2009;90(2):269–75. 19515731 10.3945/ajcn.2009.27694

[42] Zijlstra N, Mars M, de Wijk RA, Westerterp-Plantenga MS, Holst JJ, de Graaf C Effect of viscosity on appetite and gastro-intestinal hormones. Physiol Behav. 2009;97(1):68–75. 19419670 10.1016/j.physbeh.2009.02.001

[43] Weijzen PL, Liem DG, Zandstra EH, de Graaf C Sensory specific satiety and intake: the difference between nibble- and bar-size snacks. Appetite. 2008;50(2-3):435–42. 17977618 10.1016/j.appet.2007.09.008

[44] Llewellyn C, Van Jaarsveld C, Boniface D, Carnell S, Wardle J Eating rate is a heritable phenotype related to weight in children. Am J Clin Nutr. 2008;88(6):1560–6. 19064516 10.3945/ajcn.2008.26175

[45] Berkowitz RI, Moore RH, Faith MS, Stallings VA, Kral TV, Stunkard AJ Identification of an obese eating style in 4‐year‐old children born at high and low risk for obesity. Obesity. 2010;18(3):505–12. 19779474 10.1038/oby.2009.299 PMC2917041

[46] Fogel A, Fries LR, McCrickerd K, Goh AT, Quah PL, Chan MJ, Toh JY, Chong YS, Tan KH, Yap F et al. Oral processing behaviours that promote children's energy intake are associated with parent-reported appetitive traits: results from the GUSTO cohort. Appetite. 2018;126:8–15. 29551400 10.1016/j.appet.2018.03.011 PMC5973283

[47] Fogel A, Goh AT, Fries LR, Sadananthan SA, Velan SS, Michael N, Tint MT, Fortier MV, Chan MJ, Toh JY et al. A description of an ‘obesogenic' eating style that promotes higher energy intake and is associated with greater adiposity in 4.5 year-old children: results from the GUSTO cohort. Physiol Behav. 2017;176:107–16. 28213204 10.1016/j.physbeh.2017.02.013 PMC5436621

[48] Fogel A, Goh AT, Fries LR, Sadananthan SA, Velan SS, Michael N, Tint MT, Fortier MV, Chan MJ, Toh JY et al. Faster eating rates are associated with higher energy intakes during an ad libitum meal, higher BMI and greater adiposity among 4.5-year-old children: results from the growing up in Singapore towards healthy outcomes (GUSTO) cohort. Br J Nutr. 2017;117(7):1042–51. 28462734 10.1017/S0007114517000848 PMC5472197

[49] Forde CG, Fogel A, McCrickerd K Children's eating behaviors and energy intake: overlapping influences and opportunities for intervention. In: Nurturing a healthy generation of children: Research gaps and opportunities. Henry CJ, Nicklas T, Nicklaus S editors. Basel, Switzerland: Karger; 2019, p. 55–67. 10.1159/000493695 30865959

[50] McCrickerd K, Forde CG Consistency of eating rate, oral processing behaviours and energy intake across meals. Nutrients. 2017;9(8):891. 10.3390/nu9080891 PMC5579684 28817066

[51] Ford AL, Bergh C, Södersten P, Sabin MA, Hollinghurst S, Hunt LP, Hamilton-Sheild J Treatment of childhood obesity by retraining eating behaviour: randomised controlled trial. BMJ (Online). 2010;340:b5388. 10.1136/bmj.b5388 20051465

[52] Hermsen S, Mars M, Higgs S, Frost JH, Hermans RC Effects of eating with an augmented fork with vibrotactile feedback on eating rate and body weight: a randomized controlled trial. Int J Behav Nutr Phys Act. 2019;16(1):90. 31640791 10.1186/s12966-019-0857-7 PMC6805487

[53] Forde CG Flavour perception and satiation. 2nd ed. Amsterdam: Woodhead Publishing; 2016.

[54] Carmody RN, Weintraub GS, Wrangham RW Energetic consequences of thermal and nonthermal food processing. Proc Natl Acad Sci. 2011;108(48):19199–203. 22065771 10.1073/pnas.1112128108 PMC3228431

[55] Hitzmann B, Ahmad MH Measurement, modeling and automation in advanced food processing. Cham: Springer; 2017.

[56] Rolls BJ Dietary energy density: applying behavioural science to weight management. Nutr Bull. 2017;42(3):246–53. 29151813 10.1111/nbu.12280 PMC5687574

[57] Rogers PJ, Hogenkamp PS, De Graaf C, Higgs S, Lluch A, Ness AR, Penfold C, Perry R, Putz P, Yeomans MR et al. Does low-energy sweetener consumption affect energy intake and body weight? A systematic review, including meta-analyses, of the evidence from human and animal studies. Int J Obes. 2016;40(3):381. 10.1038/ijo.2015.177 PMC4786736 26365102

[58] Scrinis G, Monteiro CA. Ultra-processed foods and the limits of product reformulation. Public Health Nutr. 2018;21(1):247–52. 28703086 10.1017/S1368980017001392 PMC10261094

[59] Aguayo-Mendoza MG, Ketel EC, van der Linden E, Forde CG, Piqueras-Fiszman B, Stieger M Oral processing behavior of drinkable, spoonable and chewable foods is primarily determined by rheological and mechanical food properties. Food Qual Preference. 2019;71:87–95.

[60] Van Boekel M, Fogliano V, Pellegrini N, Stanton C, Scholz G, Lalljie S, Somoza V, Knorr D, Jasti PR, Eisenbrand G A review on the beneficial aspects of food processing. Mol Nutr Food Res. 2010;54(9):1215–47. 20725924 10.1002/mnfr.200900608

[61] Willett W, Rockström J, Loken B, Springmann M, Lang T, Vermeulen S, Garnett T, Tilman D, DeClerck F, Wood A et al. Food in the Anthropocene: the EAT–Lancet Commission on healthy diets from sustainable food systems. Lancet North Am Ed. 2019;393(10170):447–92. 10.1016/S0140-6736(18)31788-4 30660336

[62] Gustafson D, Gutman A, Leet W, Drewnowski A, Fanzo J, Ingram J Seven food system metrics of sustainable nutrition security. Sustainability. 2016;8(3):196.

[63] McCrickerd K, Forde C Sensory influences on food intake control: moving beyond palatability. Obes Rev. 2016;17(1):18–29. 26662879 10.1111/obr.12340

