# Supplementary material for: Ultra-Processing or Oral Processing? A Role for Energy Density and Eating Rate in Moderating Energy Intake from Processed Foods
Source: Curr Dev Nutr. 2020 Feb 10;4(3):nzaa019. doi: 10.1093/cdn/nzaa019 (PMC7042610; doi:10.1093/cdn/nzaa019)
Supplement: nzaa019_Supplement_Table [file nzaa019_supplement_table.docx]

**Title:** Ultra-processing or Oral Processing? A role for Energy Density and Eating Rate in Moderating Energy Intake from Processed Foods, **First Author**: Ciarán G. Forde

**Online Supplementary Material:** Energy intake rate (kcal/min) for all foods by their NOVA classification

| **Foods** | **NOVA** | **EIR (kcal/min)** | **Foods** | **NOVA** | **EIR (kcal/min)** |
| --- | --- | --- | --- | --- | --- |
| Tea | 2 | 0 | Pork fillet | 2 | 28 |
| Coffee | 2 | 1 | Orange | 2 | 29 |
| Bean sprouts boiled | 2 | 2 | Rice brown boiled | 2 | 34 |
| Lettuce iceberg raw | 2 | 2 | Beef, minced | 2 | 35.1 |
| Green papaya, shredded | 2 | 2.89 | Beef, steak | 2 | 36.4 |
| Carrot, raw | 2 | 3.52 | Grapes with skin | 2 | 37 |
| Bok Choy | 2 | 3.84 | Raisins dried | 2 | 39 |
| Carrot raw | 2 | 4 | Chicken breast, baked | 2 | 40.5 |
| Chicory boiled | 2 | 4 | Banana | 2 | 41 |
| Cucumber with skin raw | 2 | 4 | Eggs chicken boiled | 2 | 41 |
| Kailan (Chinese greens) | 2 | 4.7 | Pineapple | 2 | 42 |
| Cucumber without skin raw | 2 | 5 | Potatoes, mashed | 2 | 42.1 |
| Leek boiled | 2 | 5 | Potatoes mashed | 2 | 44 |
| Beans French boiled | 2 | 7 | Egg, hard boiled | 2 | 44 |
| Cabbage ox-heart boiled | 2 | 7 | Porridge, plain | 2 | 45.5 |
| Courgettes boiled | 2 | 7 | Beef steak tartare | 2 | 49 |
| Broccoli (steamed) | 2 | 7.2 | Cashew nuts unsalted | 2 | 49 |
| Mushroom boiled | 2 | 8 | Steak | 2 | 50.1 |
| Celeriac boiled | 2 | 9 | Chicken fillet | 2 | 56 |
| Spinach frozen boiled | 2 | 9 | Chicken breast | 2 | 58 |
| Broccoli boiled | 2 | 11 | Salmon prepared in microwave oven | 2 | 61 |
| Sweet pepper red boiled | 2 | 11 | Fish, fillet | 2 | 61.8 |
| Broccoli (boiled) | 2 | 11.2 | Kiwi fruit green | 2 | 67 |
| Tomatoes (raw) | 2 | 12.6 | Pork belly, roasted | 2 | 67.4 |
| Sotong | 2 | 13.2 | Salmon (steak) | 2 | 67.8 |
| Cauliflower boiled | 2 | 14 | Rice white boiled | 2 | 73 |
| Pear with skin | 2 | 14 | Yoghurt full fat | 2 | 76 |
| Apple, raw | 2 | 14 | Bulgour | 2 | 94.9 |
| Carrots, boiled | 2 | 14.1 | Smoothie fruit | 2 | 142 |
| Carrots (mashed) | 2 | 14.9 | Yoghurt drink | 2 | 190 |
| Potatoes without skins boiled | 2 | 15 | Milk whole | 2 | 230 |
| Ham lean boiled | 2 | 17 | Juice orange freshly squeezed | 2 | 240 |
| Tomato raw | 2 | 17 | Juice apple | 2 | 297 |
| Brussels sprouts boiled | 2 | 18 | Silver-skin onion sweet pickled | 3 | 6 |
| Apple without skin | 2 | 21 | Mushroom (canned) | 3 | 7.2 |
| Potatoes boiled with skin | 2 | 21 | Rice waffle | 3 | 9 |
| Mandarins | 2 | 22 | Gherkins sweet pickled | 3 | 12 |
| Pear without skin | 2 | 22 | Peppermint | 3 | 15 |
| Rice, brown | 2 | 22.6 | Boiled sweets | 3 | 16 |
| Apple with skin | 2 | 23 | Soup portion | 3 | 18 |
| Papaya, slices | 2 | 23.2 | Sandwich meat chicken | 3 | 22 |
| Strawberries | 2 | 24 | Baguette | 3 | 22.9 |
| Beef, steak pieces | 2 | 25.3 | Popcorn popped sweet | 3 | 24 |
| Potatos, whole | 2 | 25.4 | Tofu puffs, baked | 3 | 24.1 |
| Tomato juice | 2 | 26 | Tomatoes (canned) | 3 | 24.3 |
| Peas fresh boiled | 2 | 27 | Ham slice (THICK) | 3 | 24.3 |
| Rice, white | 2 | 27.8 | Tok | 3 | 25.3 |
| Carrot boiled | 2 | 28 | Cabbage red with apple frozen / boiled | 3 | 26 |

| **Foods** | **NOVA** | **EIR (kcal/min)** | **Foods** | **NOVA** | **EIR (kcal/min)** |
| --- | --- | --- | --- | --- | --- |
| Japanese rice cracker mix | 3 | 26 | Burger (homemade) | 3 | 68.6 |
| Prawn crackers natural | 3 | 26 | Chips fried in liquid frying fat | 3 | 74 |
| Fishball | 3 | 26.2 | Buttermilk | 3 | 80 |
| Sandwich, Hard | 3 | 27.1 | Salami Sausage Saveloy | 3 | 80 |
| Ham slice (THIN) | 3 | 27.1 | Sausage pork (Braadworst) | 3 | 80 |
| Olives ripe in brine | 3 | 29 | Chicken wings | 3 | 82.1 |
| Tofu, silken | 3 | 30 | Egg mayo | 3 | 87.4 |
| Beans brown tinned | 3 | 31 | Peanuts salted | 3 | 88 |
| Tang hoon | 3 | 32.8 | Salmon smoked | 3 | 89 |
| Biscuit salted average | 3 | 33 | Quiche | 3 | 95 |
| Ham shoulder medium fat boiled | 3 | 33 | Pasta (penne) | 3 | 98.3 |
| Ham shoulder on wholemeal bread | 3 | 33 | Cashew nuts salted | 3 | 105 |
| Chips, oven | 3 | 33.1 | Sausage smoked traditional cooked | 3 | 111 |
| Yoghurt low fat | 3 | 35 | Apple sauce | 3 | 115 |
| Apple, pureed | 3 | 36.8 | Milk semi-skimmed | 3 | 133 |
| Muesli with fruit | 3 | 37 | Cake without butter | 3 | 152 |
| Spinach creamed frozen boiled | 3 | 37 | Minced meat ball with egg/crumbs | 3 | 156 |
| Cheese Mozzarella on wholemeal bread | 3 | 38 | Breakfast drink (Goede Morgen) | 3 | 161 |
| Fromage fraise low fat | 3 | 38 | Milk skimmed | 3 | 188 |
| Tofu, firm | 3 | 40.2 | Breakfast drink (HeroFruitontbijt) | 3 | 330 |
| Bakkwa (Candied meat snack) | 3 | 41.2 | Cola light soft drink | 4 | 0 |
| Udon Noodle | 3 | 41.3 | Soup vegetable based dried packet | 4 | 9 |
| Peanuts, roasted | 3 | 41.3 | Stock from cube | 4 | 9 |
| Spaghetti | 3 | 41.9 | Crispbread wholemeal | 4 | 18 |
| Beans baked in tomato sauce tinned | 3 | 42 | Ketchup tomato | 4 | 19 |
| Herring salted | 3 | 42 | Bread brown wheat | 4 | 20 |
| Sandwich, Soft | 3 | 42.2 | Breadsticks | 4 | 21 |
| Salmon (canned) | 3 | 42.6 | Crisp-bakes Dutch wholemeal | 4 | 21 |
| Prata | 3 | 42.8 | Coffee cappuccino instant | 4 | 22 |
| Cheese Gouda 48+ | 3 | 43 | Ketchup curry | 4 | 22 |
| Muesli crunchy | 3 | 44 | Bread wholemeal | 4 | 24 |
| Rice, fried | 3 | 45.1 | Cocktail Snacks Nibbits | 4 | 24 |
| Rice, glutinous | 3 | 46.7 | Bread multigrain with seeds | 4 | 25 |
| Beer pilsner | 3 | 47 | Low fat margarine on bread | 4 | 25 |
| Pork schnitzel breaded | 3 | 49 | Soup thickened with vegetables | 4 | 25 |
| Sandwich spread on wholemeal bread | 3 | 49 | Sauce tomato readymade | 4 | 26 |
| Tortellini boiled | 3 | 52 | Soup with meat vegetables & noodle | 4 | 27 |
| Bun wholemeal with muesli | 3 | 55 | Toast | 4 | 27 |
| Steak tartare (filet American) on bread | 3 | 57 | Ice cream dairy cream based | 4 | 28 |
| Cheese 30+ | 3 | 58 | Chips, tortilla | 4 | 29.8 |
| Kueh | 3 | 59.6 | Cod prepared in microwave oven | 4 | 30 |
| Peanuts coated | 3 | 60 | Bread white water based | 4 | 32 |
| Salmon (smoked) | 3 | 60.6 | Soup vegetable readymade | 4 | 32 |
| Minced beef/pork shallow fried | 3 | 61 | Bread, white | 4 | 32.9 |
| Tuna in oil tinned | 3 | 61 | Wine-gum/fruit-gum | 4 | 33 |
| Cheese Mozzarella | 3 | 63 | Chips, potato | 4 | 33.1 |
| Chips oven frozen prepared | 3 | 66 | Chicken sliced | 4 | 33.3 |
| Cheese Brie 60+ | 3 | 67 | Crisps potato light unflavored | 4 | 37 |
| Pasta wholemeal boiled | 3 | 68 | Ice cream Stracciatella | 4 | 37 |

| **Foods** | **NOVA** | **EIR (kcal/min)** | **Foods** | **NOVA** | **EIR (kcal/min)** |
| --- | --- | --- | --- | --- | --- |
| Pineapple in syrup | 4 | 37 | Vanilla ice cream | 4 | 60.1 |
| Roll brown hard | 4 | 37 | Cake Dutch spiced (Ontbijtkoek) wholemeal | 4 | 61 |
| Roll white hard | 4 | 37 | Cheese cream soft (Boursin) on w/g bread | 4 | 62 |
| Peaches in syrup | 4 | 38 | Crisps potato light flavoured | 4 | 62 |
| Toffees | 4 | 38 | Biscuit Dutch (Krakeling) | 4 | 63 |
| Liquorice Dutch type salted | 4 | 39 | Liquorice Dutch sweet | 4 | 65 |
| Roll white soft | 4 | 39 | Chicken and prawn paella | 4 | 66 |
| Chicken ball | 4 | 39.5 | Hamburger prepared | 4 | 66 |
| Biscuit sweet | 4 | 40 | Liquorice allsorts | 4 | 66 |
| Bread pita white | 4 | 40 | Syrup apple on wholemeal bread | 4 | 66 |
| Sandwich chicken on w/g bread | 4 | 40 | Chocolate confetti plain on wholemeal bread | 4 | 69 |
| Margarine 80% fat w/g bread | 4 | 41 | Bun currant/raisin | 4 | 70 |
| Biscuit sponge fingers | 4 | 42 | Sausage luncheon meat on wholemeal bread | 4 | 70 |
| Crackers cream | 4 | 42 | Croissant | 4 | 71 |
| Roll brown soft | 4 | 42 | Chicken in barbeque sauce with potato wedges | 4 | 72 |
| Wrap/Tortilla | 4 | 42 | Fish pie | 4 | 72 |
| Biscuit fruit | 4 | 43 | Jam on wholemeal bread | 4 | 72 |
| Crisps tortilla unflavored | 4 | 43 | Peanut butter on wholemeal bread | 4 | 72 |
| Cake sponge Dutch Eierkoek | 4 | 44 | Pizza | 4 | 72.8 |
| Chocolate plain | 4 | 44 | Chicken nuggets prepared in oven | 4 | 73 |
| Biscuit brown/wholemeal | 4 | 45 | Mayonnaise | 4 | 73 |
| Bread ciabatta no filling | 4 | 45 | Spread chocolate hazelnut on wholemeal bread | 4 | 73 |
| Potato crisps oven baked | 4 | 46 | Hotdog | 4 | 73.4 |
| Soup legume based readymade | 4 | 47 | Sausage luncheon meat | 4 | 74 |
| Hot chocolate | 4 | 48 | Yoghurt low fat with fruit | 4 | 74 |
| Jell-o | 4 | 48.4 | Burger (premade) | 4 | 74.4 |
| Spring roll fried | 4 | 49 | Liver pate sausage on wholemeal bread | 4 | 77 |
| Sauce for chips 25% oil | 4 | 51 | Peanut sauce ready to eat | 4 | 77 |
| Yoghurt half fat | 4 | 51 | Lancashire hotpot | 4 | 78 |
| Cheese 30+ on w/g bread | 4 | 52 | Mushroom risotto | 4 | 78 |
| Potato slices fried | 4 | 53 | Bread white with sugar (Suikerbrood) | 4 | 80 |
| Battered fish fillet deep-fried | 4 | 53 | Mousse chocolate | 4 | 80 |
| Confetti sprinkle on w/g bread | 4 | 54 | Fish fingers | 4 | 80.3 |
| Vegetable biryani | 4 | 54 | Chocolate chip cookie | 4 | 81 |
| Biscuit fortified with currants | 4 | 55 | Energy drink (Red Bull) | 4 | 81 |
| Yoghurt vanilla half fat | 4 | 55 | Meringue cake (Bokkenpootje) | 4 | 82 |
| Biscuit spiced Speculaas | 4 | 56 | Chocolates filled/Belgium chocolate | 4 | 83 |
| Cheese spread on w/g bread | 4 | 56 | Thai green chicken curry with rice | 4 | 84 |
| Chocolate flakes on w/g bread | 4 | 56 | Biscuit Dutch shortbread sprits | 4 | 85 |
| Biscuit fortified (Liga) | 4 | 57 | Candy bar Twix | 4 | 85 |
| Cake Dutch spiced | 4 | 57 | Sausage (Frikandel) deep-fried | 4 | 85 |
| Candy bar Snickers | 4 | 57 | Waffle Luikse | 4 | 85 |
| Sausage Saveloy on w/g bread | 4 | 59 | Candy bar (Milky Way) | 4 | 88 |
| Cheese Brie 60+ on w/g bread | 4 | 60 | Garlic bread | 4 | 88.7 |
| Chicken Chow Mein | 4 | 60 | Eclair with whipped cream filling | 4 | 90 |
| Crisps potato | 4 | 60 | Pepperoni pizza | 4 | 90 |
| Fish, chips and peas | 4 | 60 | Spaghetti carbonara | 4 | 90 |
| Marsh mallows | 4 | 60 | Sweet and sour chicken with rice | 4 | 90.00 |
| Roast chicken dinner | 4 | 60.00 | Yoghurt drink with sweetener | 4 | 90 |

| **Foods** | **NOVA** | **EIR (kcal/min)** | **Foods** | **NOVA** | **EIR (kcal/min)** |
| --- | --- | --- | --- | --- | --- |
| Bacon rasher | 4 | 91 | Pancake prepared with margarine | 4 | 114 |
| Chocolate bar milk with nuts | 4 | 92 | Lasagne | 4 | 114.4 |
| Beef cannelloni | 4 | 96 | Cream whipped with added sugar | 4 | 115 |
| Chicken and bacon pasta bake | 4 | 96 | Beef stew with dumplings | 4 | 120 |
| Croissant with ham and cheese | 4 | 96 | Custard chocolate full fat | 4 | 122 |
| Croquette meat deep-fried in fat | 4 | 98 | Apple pie Dutch with shortbread | 4 | 127 |
| Pudding airy | 4 | 99 | Ice tea (sparkling) | 4 | 130 |
| Sausage frankfurter tinned | 4 | 99 | Dairy spread plain/herbs on wholemeal bread | 4 | 131 |
| Chicken nugget | 4 | 99.9 | Fromage Frais half-fat with fruit | 4 | 131 |
| Beef lasagne | 4 | 102 | Apple turnover with puff pastry | 4 | 132 |
| Macaroni cheese | 4 | 102 | Cupcake iced | 4 | 136 |
| Sausage and mashed potato | 4 | 102 | Almond paste filled tarts | 4 | 142 |
| Three cheese omelette | 4 | 102 | Cake wrapped in marzipan and chocolate | 4 | 145 |
| Roll bapao | 4 | 105 | Fruit drink concentrate diluted | 4 | 146 |
| Ice tea (non-sparkling) | 4 | 109 | Waffle syrup | 4 | 164 |
| Sausage roll in pastry | 4 | 110 | Snack sausage roll puff pastry | 4 | 182 |
| Chocolate milk | 4 | 111 | Juice orange pasteurized | 4 | 232 |
| Cream slice Dutch (Tompouce) | 4 | 113 | Milk chocolate-flavored semi-skimmed | 4 | 249 |
| Custard vanilla full fat | 4 | 113 | Milk chocolate-flavored full fat | 4 | 422 |

*NOVA Classification; 2 = Unprocessed, 3 = Processed and 4 = Ultra-processed; (w/g = whole-grain bread).*
